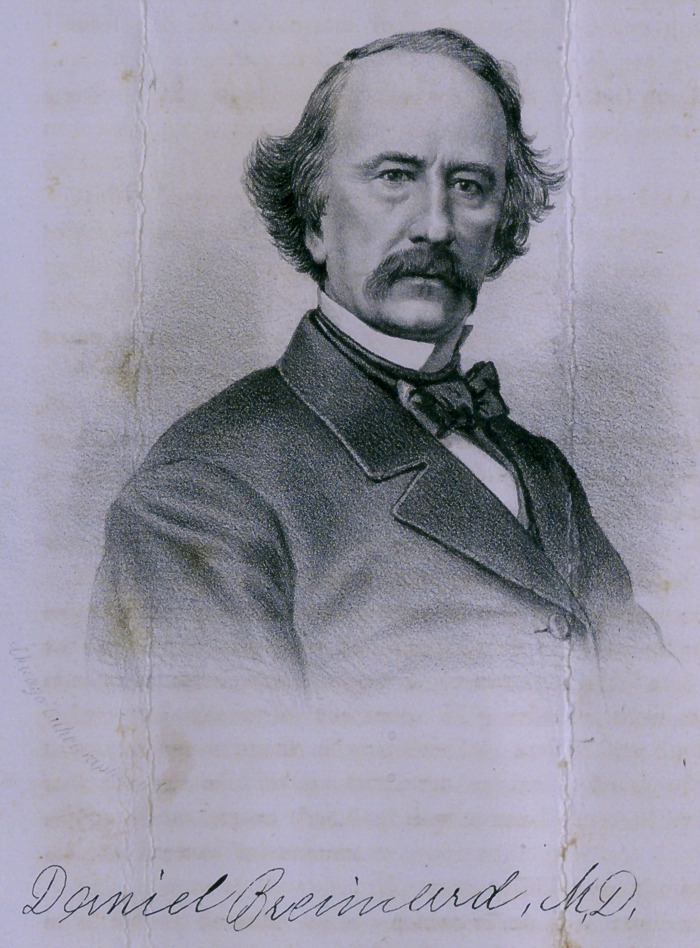# The Death of Dr. Brainard

**Published:** 1866-11

**Authors:** 


Daniel Brainard, M.D.

### THE DEATH OF DR. BRAINARD.

Prof. DANIEL BRAINARD died in this city, October 10, 1866. Less than one month previously, after a prolonged absence in Europe, he returned to his home, bringing renewed health and unwonted zeal for the prosecution of those labors which have made his name a household word throughout the country.

His health had been somewhat impaired for a year previous to his departure for Europe, but not sufficiently to prevent him from doing an ordinary amount of mental and physical labor. His disease was a functional derangement of the kidneys, diagnosticated by Trousseau and other distinguished professors in Europe as oxaluria. He spent most of his time while abroad traveling with his family in Italy and Switzerland.

The few days that intervened between his arrival and death was spent mostly at his office and in the visitation of friends. On Tuesday afternoon, October 9th, he lectured to the class at the College, devoting a part of the hour to a subject not connected with his branch—the epidemic of cholera then prevailing at Chicago. He spent the evening at his office, conversing with a number of friends. He retired at 11 o'clock with every appearance of perfect health. During the latter part of the night he had an attack of diarrhœa, which he checked by an enema of vinegar of opium. He arose the following morning and partially completed his toilette, read the morning paper, and commented on the election news—the only indication of illness being a slight moisture of the skin, which he attributed to the opium taken during the night. He took a bowl of chicken broth for breakfast, and remained comfortable until about 9 o'clock, when he was seized with a most violent paroxysm of vomiting, followed soon after by a return of diarrhœa, both of which continued at short intervals for about two hours, when they entirely ceased. During that time he sank rapidly, and by 2 o'clock was in profound collapse. He ceased to breathe at a quarter past 9 in the evening of October 10, 1866.

This melancholy event excited a profound sensation in our community. The day was dark and gloomy; the epidemic was at its height; the ensigns of mourning were overshadowing the public buildings, in memory of those officers of the city government whom the pestilence had stricken. The members of the medical profession gathered at the Court House to unite in their testimonials of respect for the honored dead, and on the morrow, a solemn assembly at St. James's Church, told how deep was the feeling of the loss which we have sustained.

It is yet too soon to discourse calmly upon the life and the character of our departed teacher. To narrate the events of that career, would be to touch upon all that pertains to the history not only of surgery, but of civilization in the Western States. Coming from the East, thirty years ago, Dr. Brainard discovered in the little town under the walls of the old fort near the river Chicago, an opening—a widening field for the exhibition of his genius. He rose at once to the leadership of his profession, and the fame of his skill outstripped even the marvelous growth of his chosen city. Twenty-three years ago, he laid the foundation of Rush Medical College, an institution which stands to-day, the proudest monument of its author's fame. Twenty-three years ago, he was the leading spirit in the management of this journal, and from that day to the present, it would be hard to find anything of value to the profession in the Northwestern States which cannot be traced directly to its source in the teeming brain of this wonderful man.

From the *Genealogy of the Brainard Family*, we quote the following sketch:

Dr. Brainard was born, May 15th, 1812, at Whitesborough, Oneida County, N. Y. He received the advantages of the Academy or High School of that town; and commenced the study of his profession there in 1829, but soon went to Rome, where he pursued them further, enjoying at the same time the benefit of lectures. He attended two courses, one at the Medical College in Fairfield, and the other at Jefferson Medical College, Philadelphia, where he was graduated in the spring of 1834. He then returned to Whitesborough, where he remained two years with his former preceptor, nominally in practice, but mostly engaged in the study of the Latin and French languages and professional teaching. In the spring of 1836, he gave his first course of lectures, which was on anatomy and physiology, in the Oneida Institute. In August, 1836, he removed to Chicago, where he remained until October, 1839, when he took a voyage across the Atlantic and visited Paris for the purpose of improving himself further for his profession, where he remained until April, 1841, when he returned and resumed his practice. Soon after this he was appointed Professor of Anatomy in the University of St. Louis, where he gave a course of lectures in 1842.

He was a Corresponding Member of the Society of Surgery in Paris, and of the Medical Society of the Canton of Geneva. His essay on the treatment of ununited fractures and deformities received the prize of the American Medical Association at its meeting in St. Louis, May, 1854. During nearly the whole of the administrations of Presidents Pierce and Buchanan he held the position of Surgeon to the Marine Hospital, and was, for a long time, Surgeon of the Mercy Hospital, etc., in this city.

February 5, 1845, he was married to Evelyn Sleight, and has had four children: Julia, Edwin, Daniel and Robert P. Brainard, the last two of which are dead.

His wife and surviving children are still in Europe.

Dr. Brainard was blessed with an iron frame, and a commanding person. His figure was tall and stately; his manner was the soul of dignity. One could not enter his presence without feeling a sense of the greatness of the man. As a teacher he stood without a rival. The order, the method, and the clearness of his lectures have never been surpassed. As a writer he is best known by the essays which have been scattered through the medical journals of the country. The great work of his life, though long announced, remains incomplete—cut short by his untimely death. As a scholar his attainments were not bounded by the limits of his profession. There was no department of science which he had not explored; there was nothing too low, nothing too high, for the range of his observation. Strength and perseverance were the pillars of his fame, and to no human power did they ever yield.

A meeting of the members of the medical profession was held at the Council Chamber, at 4 o'clock on the afternoon following the death of Dr. Brainard, for the purpose of taking proper action in relation to the sad event.

On motion, Dr. C. H. Duck was elected Chairman, and Dr. Charles G. Smith, Secretary.

The Chair then stated that it was the object of the meeting to pay the last sad tribute to a departed brother.

Dr. Brock McVickar rose and said:

The occurrence which has drawn us together has been recited in brief and emphatic words in our city papers to-day, "Died, October 10th, 1866, of cholera, Daniel Brainard, M. D., aged 53 years." Gentlemen, when we last assembled in this hall, as members of the profession to which we belong, we came together to record our respect and consideration for a departed brother, who had been taken away from us in the spring time of professional life, with the future all bright and promising before him. To-day we come to perform the same sad service for another; one older in years and full of honors, one whose ambition and aspirations for the future had become to him the fruitions of the present, one who stood, with well-earned laurels, at the head of the profession which he dignified and adorned. Gentlemen, Daniel Brainard was no ordinary man. He was possessed of sterling abilities, quickened and developed by culture and study; of untiring industry; of unremitting perseverance and unflagging zeal in the pursuit of knowledge. He was cold and reticent at times, but what seemed coldness to many was but the absorption of a strong and earnest nature, pressing forward to the attainment of the high mark he had proposed to himself. He had an abiding hatred for pretenders and shams, and looked with little favor upon those who followed their profession without seeking to rise above its daily routine of duties. When once satisfied of a man's integrity, earnestness and intelligent devotion to his profession, he was his friend. With Dr. Brainard, with but slight interruption, my relations, personal and professional, have always been of the most agreeable character; and at the time of his death, they were particularly so. I shall never forget how, during my last interview with him, a few days since, I was impressed with respect and regard for him as a gentleman, courtly in his manners, polished by foreign travel and culture, the peer of the noblest in the land. His example, Gentlemen, may serve to illumine the pathway of the student, to quicken him to higher and better efforts, and give him assurance of success. Of Dr. Brainard's position, jealousy has been manifested by smaller minds, many times falling under my own knowledge. He was restless sometimes under this jealousy and its resulting injustice, and that restlessness, at an early day, toned and tempered his feelings; but, when a man has attained the measure of success which fell to his lot, and which he earned so faithfully, he can afford to smile, instead of being annoyed, and look down, as he did ultimately, with the calm consciousness of superiority, obtained by his own efforts and the exercise of the powers and faculties given him by God. But he is gone from us, and the places which knew him once shall know him no more. Peaceful be his rest, green be the turf which grows above him, and bright be the place which he shall hold in the memory of friends he has left behind.

I move you, sir, the appointment of a committee of five to draft resolutions expressive of the sense of this meeting on the occasion of the decease of our brother, Daniel Brainard.

This motion being carried by a unanimous vote, the Chair appointed, as such committee, Doctors McVickar, H. A. Johnson, Trimble, Charles G. Smith and G. C. Paoli.

The committee subsequently reported the following:

*Resolved*, That in the dispensation of Divine Providence, which has removed from our midst our deceased friend and brother, Daniel Brainard, we recognize the hand of Him who does everything well, and bow submissively to His will.

*Resolved*, That our deceased brother, by his natural powers of mind, by his capacity as a teacher, by his untiring industry, by his unwearied zeal and assiduity in the profession of which he was a distinguished member and ornament, has acquired a position of character and usefulness, recognized not only by his colleagues at home, but by the profession throughout the world.

*Resolved*, That in his example we read the great lesson of encouragement in the paths of duty and honor, and while humbly seeking to imitate it, we hold it up to the consideration of members of his own and other professions everywhere; and that in his death the profession of our city and the Northwest has suffered an irreparable loss.

The resolutions were adopted.

Dr. G. C. Paoli paid the following tribute to the memory of the deceased:

There is a certain sadness and solemnity in our meeting to-day, to pay our respect to our departed friend and professional brother.

The profession deeply feel the loss they have sustained in Professor Brainard's death, for he was no ordinary man. Highly gifted by nature, his powers were cultivated by study, and from pure love of the profession, he devoted himself with untiring zeal to the work of instruction. As a surgeon, he had few equals—as an operator, he was cool, cautious and bold.

As a lecturer, he possessed, to a remarkable degree, the rare talent of profound clearness in communicating his ideas to his listeners, and the most difficult subject in surgery he always imparted to the student with a certain plainness and simplicity, and excelled all other lecturers whom I have heard in condensing the greatest amount of instruction in the fewest words.

He was an acute observer of nature, which made him, as a lecturer, at once practical and original. Rush Medical College possesses several eminent men, but Brainard stood foremost among them. His reputation also stands high abroad, and though he had lived long enough to win fame and honor, his life was all too short for humanity. His teachings belong to the future, and surgical annals will place him amongst the greatest surgeons of the nineteenth century.

On motion of Dr. Johnson,

*Resolved,* That a committee of five be appointed to take measures to procure a marble bust, or some other permanent memorial, of the late Dr. Brainard.

The resolution was carried, and the following gentlemen were appointed the committee: Dr. R. C. Hamill, Dr. H. A. Johnson, Dr. J. V. Z. Blaney, Dr. N. S. Davis, Dr. DeLaskie Miller.

At the request of Dr. Powell, nephew of the deceased, six of the twelve pall-bearers were selected by the meeting. They were as follows: Drs. Hitchcock, Duck, Eldridge, Hamill, Paoli and Johnson.

At a meeting of particular friends, the following distinguished citizens were also selected as pall-bearers: His Honor the Mayor, J. B. Rice, Hon. Charles Walker, Hon. J. Young Scammon, John L. Wilson, Esq., Dr. C. V. Dyer, and Julian S. Rumsey, Esq.

---

At a meeting of the Faculty of Rush Medical College, held at 12 o'clock of October 11th, to receive the announcement of the death of Prof. Daniel Brainard, M. D., President of the Faculty and Professor of Surgery, the following resolutions, reported by a committee of their number, were unanimously adopted:

WHEREAS, It has pleased Divine Providence to remove, by the hand of death, our revered President, Daniel Brainard, M.D.;

*Resolved,* That in his death we, as a Faculty, have sustained a shock which we feel that words are inadequate to express.

*Resolved,* That we mourn his loss as of a colleague, a brother, a friend—and a chief—a loss that we feel to be irreparable, not only to ourselves but to our alumni, our class, and to the profession of the world.

*Resolved,* That in the death of Daniel Brainard, the profession at large has lost one of its most distinguished members, and one of the most useful and devoted of its co-laborers, and that his contributions to its advancement and progress, will perpetuate his memory in the history of the profession.

---

The funeral ceremonies were conducted at St. James' Church, cor. of Huron and Cass streets, by Rev. Clinton Locke, Rector of Grace Episcopal Church. The occasion was a solemn and impressive one, and as such it was felt by all who were present. It was apparent that the sudden death of this honored and eminent man had cast a gloom not only upon those of his profession, but to a certain extent over the whole city.

At 10 o'clock a number of the friends of the deceased met at the residence of Colonel J. H. Bowen, on Michigan avenue, from whence they proceeded to the church to receive the remains.

At 11 o'clock the coffin was taken from the funeral car by the pall-bearers, and carried into the church. In the porch stood ladies, each with a floral wreath or immortelle, which they laid upon the bier as it passed. At the same time the clergyman proceeded to read the burial service, commencing—"I am the resurrection and the life." The coffin was borne along to the front of the altar, followed by the members of the medical profession in a body, and the students of the College.

After the usual prayers and the reading of the Scriptures, Rev. Clinton Locke delivered the following funeral discourse:

Well does Horace say that "gloomy and impartial death knocks at the door of the royal palace as well as at the door of the cabin of the pauper." Our Last Enemy, as the Scriptures expressively call him, is not daunted by station, nor careful of feelings. He would enter a congress of the world's monarchs, lay his hand upon the shoulder of its mighty head, and perforce, the stricken one must lay aside the crown and sceptre, pomp and power, and follow him. He is utterly regardless of the crying need there may be for a man, his brilliant acquirements, the usefulness of his position, the void his death will create; relentless, immovable as a heart of marble, he moves among men and summons them away. Mediæval tradition loved to represent him under the figure of a woodman walking through a forest, now cutting down a tender sapling, now a hard and sturdy oak, now cropping a just opening flower, and now overturning an aged tree tottering in the wind. Cruel and mysterious being, we shudder when we think of him and all his loathsome adjuncts, and indeed the misery and the wretchedness he causes would be without one single ray of comfort if the eye of faith did not see standing beside his skeleton the all-glorious figure of the Lord of the Resurrection, bearing the trophies of his victory over death, pointing to the stone rolled away from the sepulchre, and proclaiming that "Christ being raised from the dead, death hath no more dominion over him; that as in Adam all die, even so in Christ shall all be made alive." This alone can enable us to think of death with composure. His victory is only for a time. He cannot separate us for eternity from those we love. He cannot prevent our individuality from vesting itself in the mystic garment of its celestial state, and living again forever, invulnerable to any dart of his. We can heap the mound over the coffins of our dearest ones, with a firm hope below all our tears that no power can kill the soul; deathless, immortal, it is already in the place of departed spirits, awaiting the judgment; already, if it has been faithful, enjoying a foretaste of the blessedness of Heaven; and if faithless, the premonitions of supreme despair.

Busy, indeed, my friends, is the woodman death now among us. Fast and thick his strokes fall upon the trees of the forest, and while for all we feel a general sorrow, yet, when there comes crashing down one of the noblest oaks in all the woodland, whose shaft had pushed up so high, whose branches spread out so wide, we cannot help pausing a moment to grieve over the loss, and admire the beautiful proportions. The simile is not out of place, I feel, at the funeral service at which you are now assisting. The presence of so many well known public men, so many of the first members of the medical staff of the city, even if I were totally ignorant of the name and fame of the tenant of this narrow house, would assure me that this was no common death, no every-day bereavement. But this crowd of honorable and distinguished mourners was not needed for that. The dead before us had not only a Chicago, but an American, not only an American, but a foreign reputation. He had earned for himself the foremost place in his peculiar department in all this vast Northwest. He took rank with such men as Parker, Post and Gross, and when in after times some chronicler gathers up the names of the most distinguished surgeons, the name of Brainard will be found the peer of Astley Cooper, John Hunter, and the elder and younger Larreys. With the date and place of his birth, and the circumstances of his education, the daily press has made you familiar. He did not, like so many, consider that when he left the lecture-room, to assume the M.D., his studies were over, and he perfectly competent to treat everything from a felon up to a compound fracture; but throughout his long life he availed himself eagerly of every opportunity of instruction. The great schools of Paris knew no more assiduous student, and he had just returned from another visit to those splendid centres of medical learning which the experience of centuries has built up abroad. It was in the branch of surgery that our departed friend gained his first laurels. His favorite department, he loved it to the end with all the ardor of a young enthusiast. He was a brilliant operator, firm in touch, rapid in movement, never flurried by even the most trying crises, perfectly conversant with what was to be done and how to do it. Possessing beyond the methods of the schools that inventive faculty, that power of combination which alone can make a man great, he had risen to the very head of his profession, and what is unusual, he had amassed a large fortune by his professional exertions, a much rarer thing than persons outside the medical profession are apt to think. Not only as a surgeon, however, did he merit distinction, but as a man of science, particularly in the departments of natural history, anthropology, botany and geology. He was an authority in all these points, and amid all the cares of his immense practice, kept himself freshly informed of the latest discoveries in these sciences so near akin to his own profession. He was, moreover, a man of extensive general information, of fine literary taste, well informed, and thoroughly interested in the commercial and municipal relations of this city, with which he has ever been identified. His taste and acquaintance with sculpture and painting was that of a thorough connoisseur, and he has left among his most cherished possessions works of art of the first class, of which Chicago may well be proud. His greatest monument will be the college of which he was the founder. Standing in its halls and asking of Brainard, the answer might well be the epitaph of Sir Christopher Wren—"*Si quæris monumentum circumspice.*" It may be out of place, but I cannot help suggesting that henceforth that institution should bear his name, that legal enactment should give a right henceforth to say, "Brainard Medical College."

To strangers he may have seemed cold and reserved, but it was because he had no time for trifling, or for the bald common-places of society. His friends never found him otherwise than genial, polished and ready with his counsel and his aid. Many a young man among the twelve hundred who have come under his care, knows how freely his purse was open to his necessities, for he was a generous, cheerful, open-handed giver, and he took above all the greatest delight in helping onward a struggling youth, desirous, and yet pecuniarily unable to acquire the necessary information. One lesson his life teaches which should sink deep into the hearts of all the younger members of his profession. He gained his place by the hardest, the most exhausting labor. He may have had natural genius, but he looked for success to application, and he was never for one moment idle. He was not, what I wish I could say of him, a devout Christian, but he was no disregarder of religion, no scoffer at its doctrines, and he was far from having those materialistic views so common with many of his brethren. The Bishop of the Diocese was for years his intimate friend, and my place would have been his if he could have been here.

He is gone. Suffering humanity will feel the void. The great and noble profession he so adorned will miss his voice, the college of his love will sorrow over its bereavement, a far-off family will feel a double desolation when the sad news of a husband and father's death reaches them amid a strange city; and we stand silent and humbled when we think that he, who had met and conquered death so many times for others, who was fully armed with every weapon which could ward off from man the arrows of the destroyer, to whom so many grateful hearts among poor and rich turn as their preserver from a premature grave, should have been forced to throw down his arms, and in a few swift hours to bend his neck to the blow of relentless death. How truly it shows the vanity, the nothingness of human knowledge.

At the conclusion of the address, the remains were taken back to the funeral car, and the cortege, composed of about thirty carriages, proceeded to the old City Cemetery, where the remainder of the funeral service was read. The remains were then placed in the vault, where they await the wishes of the friends of the deceased.

A special meeting of the Sangamon County Medical Society was held in the city of Springfield, October 13th, at 3 P. M., Dr. Wright, of Chatham, Vice-President, in the Chair.

On motion of Dr. Wardner, the Chair appointed a committee of three to draft suitable resolutions regarding the death of Professor Daniel Brainard, of Chicago. The committee consisted of Drs. Wardner, Griffith and Bailhache.

On motion of Dr. Townsend, Dr. Wright was added to that committee.

The committee reported back the following preamble and resolutions, which were unanimously adopted:

WHEREAS, It has pleased an all-wise Providence to remove by death, Daniel Brainard, M.D., late President and Professor to Rush Medical College, whose ability and professional attainment gave him high rank in medical science, not only among friends and acquaintances, but among all devotees of the science he so much honored; therefore,

*Resolved*, That in the death of Dr. Brainard, the profession is bereft of an able teacher, science of an ardent student, and the community in which he lived of a valuable citizen.

*Resolved*, That we tender to the Faculty of Rush Medical College, our condolence for the loss of its President, and one of its most honored teachers.

*Resolved*, That we tender to the afflicted family our sympathies in this, their hour of sad bereavement.

*Resolved*, That the Secretary furnish a copy of these resolutions to the family of the deceased, and the Faculty of Rush Medical College; also, a copy to each of the papers of this place, and to the CHICAGO MEDICAL JOURNAL and *Chicago Medical Examiner*, for publication.

N. WRIGHT, *Chairman*.

A. L. CONVERSE, *Sec.*

---

NEW ORLEANS, Oct. 22, 1866.

DR. DELASKIE MILLER:

*Dear Sir*,—By your favor of the 15th inst., I learn with profound regret the death of Prof. Brainard, a name dear to science—an ornament to our profession.

In labors great, in knowledge deep,
His work well done—let Brainard sleep;
Erect his tomb hard by the lake,
Where waves on waves resounding break,
And chant upon the shelly shore
His *requiem* forever more.

BENNET DOWLER.

## Figures and Tables

**Figure f1:**